# Pathogen Webs in Collapsing Honey Bee Colonies

**DOI:** 10.1371/journal.pone.0043562

**Published:** 2012-08-21

**Authors:** R. Scott Cornman, David R. Tarpy, Yanping Chen, Lacey Jeffreys, Dawn Lopez, Jeffery S. Pettis, Dennis vanEngelsdorp, Jay D. Evans

**Affiliations:** 1 Bee Research Laboratory, Agricultural Research Service, United States Department of Agriculture, Beltsville, Maryland, United States of America; 2 Department of Entomology, North Carolina State University, Raleigh, North Carolina, United States of America; 3 Department of Entomology, University of Maryland, College Park, Maryland, United States of America; Baylor College of Medicine, United States of America

## Abstract

Recent losses in honey bee colonies are unusual in their severity, geographical distribution, and, in some cases, failure to present recognized characteristics of known disease. Domesticated honey bees face numerous pests and pathogens, tempting hypotheses that colony collapses arise from exposure to new or resurgent pathogens. Here we explore the incidence and abundance of currently known honey bee pathogens in colonies suffering from Colony Collapse Disorder (CCD), otherwise weak colonies, and strong colonies from across the United States. Although pathogen identities differed between the eastern and western United States, there was a greater incidence and abundance of pathogens in CCD colonies. Pathogen loads were highly covariant in CCD but not control hives, suggesting that CCD colonies rapidly become susceptible to a diverse set of pathogens, or that co-infections can act synergistically to produce the rapid depletion of workers that characterizes the disorder. We also tested workers from a CCD-free apiary to confirm that significant positive correlations among pathogen loads can develop at the level of individual bees and not merely as a secondary effect of CCD. This observation and other recent data highlight pathogen interactions as important components of bee disease. Finally, we used deep RNA sequencing to further characterize microbial diversity in CCD and non-CCD hives. We identified novel strains of the recently described Lake Sinai viruses (LSV) and found evidence of a shift in gut bacterial composition that may be a biomarker of CCD. The results are discussed with respect to host-parasite interactions and other environmental stressors of honey bees.

## Introduction

In addition to producing hive products such as honey, bee pollen, and propolis, managed colonies of the European honey bee, *Apis mellifera,* are in increasing demand for commercial crop pollination [1], [2] Yet in the midst of this demand, beekeepers on multiple continents have suffered severe losses in recent years [3], [4]. Since 2006, a substantial fraction of honey bee losses in the United States have been ascribed to Colony Collapse Disorder (CCD), an enigmatic sudden disappearance of adult worker bees [5]. ‘Disappearing diseases’ similar to CCD have long been described in honey bees, and are apparently a recurring feature of domesticated honey bee populations. Historically, these declines have not shown recognized pathologies [6] and have generally gone unresolved for years following their occurrence [7], [8].

Current research on this phenomenon has focused on three general, non-exclusive factors: (1) environmental contaminants, especially agricultural pesticides; (2) poor nutrition and subsequent developmental disorders; and (3) novel or resurgent pathogens. While numerous additional hypotheses have been raised to explain CCD, including genetic homogeneity, breakdowns in social cues, a failure in colony thermoregulation, and the impacts of genetically modified or toxic pollen [9], [10], these hypotheses have not found broad support in studies to date.

Current evidence for a chemotoxic basis of CCD is equivocal. Honey bees have been exposed for many years to diverse anthropogenic chemicals, primarily agricultural applications aimed at reducing pest plants or arthropods. Chemical residues, including known insecticides, have been detected in bees and in hive materials (mostly wax and pollen) [11]. Recent evidence suggests the effects of low-level exposure to such chemicals range from impaired behavior (Henry et al., 2012) to lowered disease resistance (Alaux et al., 2012, Pettis et al., 2012), and further study of agrochemical toxicity is warranted. Nevertheless, neither individual chemicals nor overall chemical loads have been tied to increased risk of CCD; in fact, levels of the pesticides coumaphos and Esfenvalerate have been found at higher levels in control colonies as compared to CCD colonies [5], [10]. The interpretation of this finding is complicated by the fact that coumaphos is itself directly applied to honey bee hives to reduce levels of the parasitic mite, *Varroa destructor*, and thus the apparent positive correlation between coumaphos level and colony health is confounded with *Varroa* management practices. Even so, genes presumed to be involved in pesticide detoxification have not been detected as differentially expressed in bees from CCD versus non-CCD colonies [12].

While nutritional resources certainly affect honey bee longevity, including survival over the stressful winter (when CCD has been most prevalent), there is no direct evidence linking food resources to colony collapses. Bees from collapsed colonies showed typical body weights, protein complements, and lipid stores when compared to temporal controls [5].

There are many microorganisms that affect honey bees, ranging from viruses to bacteria, fungi, trypanosomes, and amoebae [13], [14]. The roles of many of these microbes on individual and colony health remain unclear, and even less understood are the interactions and relationships among pathogens. In an earlier microbial survey in the U.S., declining honey bees colonies showed an especially high prevalence of two dicistroviruses, Israeli acute paralysis virus (IAPV) and Kashmir bee virus (KBV), and two microsporidian species in the genus *Nosema* when compared to healthy controls [15]. Of these, IAPV was most strongly linked to colony collapse, a trend that has not been supported with deeper sampling effort [5]. Nosemosis has since been associated with collapsing hives in additional studies [16], but other work has not found *Nosema* to be a predictor of CCD [5] or general colony loss [17] and the broad distribution of *Nosema* in apparently strong colonies [18], [19] contradicts a unifying role for these pathogens in CCD. Moreover, no consistent differences were observed in the expression of honey bee immune genes between CCD and non-CCD samples [12], indicating the absence of a characteristic immune response associated with this syndrome. However, CCD colonies did have more pathogen species present than did non-CCD colonies in a recent survey and there was evidence that the condition is contagious [5]. Furthermore, some viruses have been found to be predictors of overwinter colony loss [17], and an increase in ribosomal RNA fragments among transcripts from CCD samples was interpreted as implicating one or more of a group of honey bee RNA viruses [12]. Taken collectively, current data suggest that multiple factors underlie CCD, some of which may be interchangeable or dispensable but which may interact synergistically to cause disease. Thus, while a prominent role for pathogens seems likely, the causes of CCD remain elusive.

Here we further explore the connections between pathogens and CCD via a country-wide survey of pathogens in collapsed and healthy colonies. This survey includes but expands upon samples previously collected and analyzed [5], [15]. A retrospective strategy is an efficient approach to identifying potential pathogen interactions associated with CCD and a necessary prelude to laborious and costly prospective studies specifically targeting this syndrome, given the erratic nature of its occurrence. For example, a recent, large-scale prospective study of honey bee colonies over a ten-month period provided an invaluable catalog of pathogens in managed colonies but did not encounter any unexplained colony losses [19].

Our survey was based on two approaches. We first used quantitative real-time PCR (qPCR) assays for the major honey bee pathogens to provide a fine-scale analysis of pathogen levels in individual bees, colonies, apiaries, and the U.S. as a whole. Our objectives were to 1) further quantify pathogen loads in CCD colonies (relative to non-CCD colonies) across a broad geographic area; 2) identify significant covariation among pathogens and determine if such correlations are greater in CCD colonies. We then used deep sequencing to identify novel microbial strains and species, and to compare levels of the predominant gut microbiota in bees from CCD and healthy colonies. As adult honey bees have a relatively simple and consistent gut microbiome [15], [20], deviations in this flora could be a biomarker for, or directly related to, disease.

We found that CCD colonies exhibited a higher prevalence, abundance, and positive covariance of pathogens. In marked contrast, otherwise weak colonies lacking CCD traits did not have increased pathogen loads relative to strong colonies, and non-CCD colonies in general exhibited little pathogen covariance. It remains unknown whether these statistical associations reflect an actual synergy among pathogens in CCD hives or are instead ancillary to some other variable, but we show that positive correlations among pathogens develop at the level of individual bees and are not contingent on the pre-existence of CCD. We also found important heterogeneity in pathogen distributions in our samples, which were collected from across the United States. While our data supports the view that pathogen interactions contribute to CCD, they also indicate that there may be multiple routes to the same phenotype or that particular combinations are not deterministic in their effects. Finally, we take a metagenomic step toward elucidating other biotic components of CCD by identifying novel virus strains and finding evidence of a CCD-associated shift in gut bacterial composition.

## Materials and Methods

### Colony Censuses and Collection of Material

CCD and some non-CCD colonies were sampled from the same apiaries in late 2006 and early 2007 as described in [5], [15]. CCD cases were drawn either from temporary migratory commercial beekeeping apiaries on the East Coast (n = 24) or temporary commercial apiaries established in California for almond pollination (n = 37). The latter had previously been stationed in Minnesota, Pennsylvania, Washington, Nebraska, and Montana. We sought additional non-CCD colonies that were far from regions with CCD, out of concern that some non-CCD colonies could potentially have developed CCD at a later date (this was not monitored). The non-CCD samples can therefore be subdivided into ‘sympatric’ colonies that were in or near CCD outbreaks (n = 37) and additional ‘non-sympatric’ colonies (n = 26) that were taken from California apiaries with no record of CCD in January, 2008, and similarly healthy Maryland apiaries in July, 2004, and July, 2008. As a result, there is both temporal and geographic breadth in our sampling but we were not able to explicitly pair CCD and non-CCD colonies for each sampling time and location. All sampled colonies were ‘overwintered’, i.e., hives that were, in the view of their managers, healthy the previous summer and provisioned adequately for winter. A detailed analysis of how CCD and non-CCD hives differ at diagnosis in terms of population size and age structure is given in [5]. For each colony, we collected over 200 live adult bees from the nest interior and shipped these in 50 ml centrifuge tubes on dry ice. After shipping, samples were stored at −80°C until analysis.

### Estimates of Pathogen Abundance: Among-colony Analysis

Abdomens were cut from eight bees from each colony and ground together in 4 ml RNAqueous buffer (Ambion). Whole bees were not used because of the potential for PCR inhibition [21]. A 700 µl aliquot of this homogenate was extracted according to the manufacturer’s protocol. cDNA was generated with Superscript II (Invitrogen) and primed by an oligo-dT cocktail 12–18 nt in length.

qPCR reactions were carried out in 96-well plates using a Bio-Rad iCycler (Bio-Rad Corp). Reactions consisted of 1.5 µg template, 1 U Taq with proscribed buffer (Roche Applied Sciences), 1 mM dNTP mix, 2 mM MgCl_2_, 0.2 µM of each primer, 1X concentration SYBR-Green I dye (Applied Biosystems), and 10 nM fluorescein in a 25 µl reaction volume. All reactions were carried out with a thermal protocol consisting of 5 min at 95°C, then 40 cycles of a four-step protocol consisting of 94°C for 20 s, 60°C for 30 s, 72°C for 1 min, and 78°C for 20 s. Fluorescence measurements were taken repeatedly during the 78°C step to reduce the contribution of primer artifacts to the inferred concentration of the target. Dissociation kinetics were monitored to verify the product melt temperature, and a subset of products for each targeted pathogen was sequenced to confirm primer specificity [22]. Positive and negative control reactions were run on each plate.

We used published primers (Table 1 of [5]) to survey for nine known honey-bee pathogens: KBV, IAPV, deformed wing virus (DWV), acute bee paralysis virus (ABPV), black queen cell virus (BQCV), sacbrood virus (SBV), *Nosema ceranae*, *N. apis*, and the trypanosome *Crithidia mellificae*. Given the past importance of IAPV as an indicator of bee disease, three primer pairs [5] were used to confirm the sensitivity of this assay. (One primer listed in [5] was discovered to be incorrect; the actual sequence for IAPV-PW-R17 is GCAGGACATTAATGTACTATATCCAG). The mean amplification efficiency of each qPCR primer pair (**File S1**) was estimated by dilution-series analysis. The geometric mean of three honey-bee genes – actin, ribosomal protein S5 (RPS5), and microsomal glutathione-S-transferase (MGST) – was used to calculate the normalized abundance of each target in each sample (ΔC_T_), following the recommendation of [23]. Since the mean of multiple efficiency estimates for each control gene was close to one, we assumed equal and perfect efficiencies of the reference genes. However, we used the actual estimated efficiency for each target primer pair to calculate fold change in abundance, using the ΔΔC_T_ method [23]. That is, differential abundance equals (1+efficiency) ^ΔΔCT^, where ΔΔC_T_ is the difference in mean ΔC_T_ between two sample populations.

**Table 1:** Honey-bee pathogen incidence by colony status.

Pathogen	Present,non-CCD colony	Absent,non-CCD colony	Proportion present, non-CCD colony	Present,CCD colony	Absent,CCD colony	Proportion present, CCD colony	P-value
ABPV	30	33	0.48	31	30	0.51	0.722
BQCV	53	10	0.84	55	6	0.90	0.314
DWV	26	37	0.41	38	23	0.62	0.019
IAPV	10	53	0.16	15	46	0.25	0.225
KBV	8	55	0.13	23	38	0.38	0.001
SBV	9	54	0.14	16	45	0.26	0.096
NC	36	27	0.57	41	20	0.67	0.247
NA	6	57	0.10	20	41	0.33	0.001
*Crithidia*	49	14	0.78	53	8	0.87	0.182

Values are the number of colony samples (n = 61 for CCD and n = 63 for non-CCD) in which the pathogen was detected at any level. The likelihood ratio chi-square test of contingency was used to compute the probability of equal pathogen incidence in CCD and non-CCD colonies. ABPV = acute bee paralysis virus; DWV = deformed wing virus; SBV = sacbrood virus; BQCV = black queen cell virus; IAPV = Israeli acute paralysis virus; KBV = Kashmir bee virus; NC = *Nosema ceranae*; NA = *Nosema apis*.

Whether the mean difference (ΔΔC_T_) in pathogen abundances was significant was determined by Analysis of Variance of ΔC_T_ without conversion to linear relative abundance for each sample, because converted values can have strongly non-normal distributions, particularly for viruses. In addition to comparing CCD colonies to non-CCD colonies, we performed an additional analysis that decoupled the effects of colony size (an indicator of colony strength [5]) from CCD *per se*. Colonies from apiaries that had no report of CCD were split into ‘weak’ colonies with six or fewer frames of bees (n = 15) and ‘strong’ colonies with seven or more frames of bees (n = 29). These thresholds correspond to a natural break in colony size distribution, while retaining sufficient samples in each bin for statistical analysis. Partial correlations of abundance of each pathogen were estimated using Spearman’s rho statistic with a Bonferroni correction for multiple tests.

### Pathogen Covariance in Individual Bees

We investigated whether covariation in pathogen abundance extended to individual bees with natural infections of common pathogens. We quantified pathogen loads of individual workers taken from otherwise strong colonies known to contain *N. ceranae*. This analysis was deliberately removed from the context of CCD, and focused on *Nosema* and several RNA viruses because prior work had already suggested synergistic interactions among these common pathogens [15]. We sampled 17–24 adult workers from each of four colonies located in Raleigh, North Carolina (n = 77 bees). qPCR was performed for Chronic bee paralysis virus (CBPV), BQCV, DWV, *N. apis*, and *N. ceranae*. (Note that CBPV was not included in the survey of CCD and non-CCD colonies because initial work and subsequent RNA sequencing indicated a very low incidence/abundance of this pathogen in those samples.) Total RNA was isolated from the thorax using the RNeasy Mini kit and cDNA synthesis performed using 3.0 µl buffer, 3.0 µl 2.5 mM dNTP, 0.75 µl RNaseOUT, 0.3 µl (0.18 µg) of random primer cocktail, 0.75 µl Superscript III, 2.2 µl H2O, and 5 µl (approximately 2.5 µg) RNA. qPCR measurements were performed on an ABI 7900 Fast Real-Time PCR system with Sequence Detection Systems software version 2.3. qPCR reactions included SYBR Green Master Mix, 10 picomoles of each primer, and 2 µl of cDNA in a 10 µl volume. Product specificity was evaluated by dissociation curve. Total loads and partial correlations were calculated as above, except with RPS5 as the single normalization gene.

### Deep RNA Sequencing of Healthy and Collapsed Colonies for Microbe Discovery

RNA was pooled by combining equal aliquots from each CCD or non-CCD colony described above. Five µg of RNA from the “CCD−” pool was used to generate cDNA using a cocktail of random heptamer primers. cDNA was size-selected from agarose and end-polished with End Repair Enzyme (Illumina) following manufacturer protocols. A 3′ polyadenine tract was then added with Klenow fragment (Invitrogen) and the products purified with a Qiaquick DNA purification column (Qiagen). Illumina adapters were ligated to cDNA with T4 DNA ligase and the products were amplified under the following thermocycler conditions: an initial denaturing step at 98°C for 30 seconds, followed by 14 cycles at 98°C for 30 seconds, 65°C for 30 seconds, and 72°C for 30 seconds. Final products of 100–300 bp were size-selected from agarose and sequenced on an Illumina Genome Analyzer by the Institute for Genome Sciences, University of Maryland, Baltimore.

Equivalently prepared cDNA from the “CCD+” pool was sequenced using a paired-end strategy with a 350-bp fragment size. A paired-end approach facilitates the assembly of longer contigs, and therefore may provide more diagnostic sequences for annotation, but at a cost of reduced read length (67 bp). Both sequencing runs were quality-trimmed by retaining only the longest contiguous sequence of each read with a minimum (Phred-equivalent) quality score of 15, excepting at most one ambiguous base. Reads less than 50 bp after this trimming step were discarded. A small number of reads were removed because they matched Illumina primer sequence in the Univec database (www.ncbi.nlm.nih.gov/VecScreen/UniVec.html).

Reads were assembled into contigs using the Velvet assembly package [24]. CCD− reads were assembled into contigs using multiple iterations of Velvet with successive hash lengths of 21, 31, 41, 51, or 61. Contigs of less than 100 bp or with less than 3X coverage were discarded. This assembly strategy was chosen to accommodate the broad spectrum of RNA sources in the sample (viruses, a diverse bacterial community, and eukaryotic pathogens as well as the host genome) that are likely to have different optimal hash lengths for assembly. CCD+ reads were assembled in a similar fashion without read-pair information; in addition, a single paired-end assembly was performed with Velvet using a hash length of 21 and an expected fragment length of 350. Contigs from all intermediate assemblies were then merged using the BlastClust component of BLAST at 98% identity and 90% nonreciprocal overlap. Because there was substantial redundancy of contigs remaining after this step, we input the contigs to CAP3 [25] for more aggressive assembly, requiring a 60-bp overlap with 92% identity. Raw reads are available as accessions SRX028143 and SRX028145 of the NCBI Short Read Archive, however, the resulting contigs were not submitted because of an NCBI policy against hosting assemblies from mixed sources. The contigs are included here as **File S2**.

Contigs were searched against the GenBank nr database with BLASTN and BLASTX, requiring an expectation of ≤1.0E-10. All ribosomal matches to trypanosomes (e.g., *Leptomonas* and *Leishmania*) were considered to be *Crithidia mellificae* for this analysis, although the taxonomy of bee trypanosomes is not well established [26] and there is evidence of substantial genetic divergence among isolates from honey bee (R. Schwarz and J. DeRisi, unpublished data). Contigs with BLASTN hits to bacterial ribosomal sequence were submitted to the Classifier tool [27] for taxonomic evaluation. In addition to these bacterial contigs, we also used GenBank accessions of honey bee gut bacteria for read mapping (see below) because they are cloned 16S amplicons rather than short-read assemblies and are also longer on average than our contigs.

We used Bowtie [28] to map reads to reference sequences for quantitative comparisons between CCD− and CCD+. To avoid an ascertainment bias between the two sequence samples, which have different maximum lengths, we trimmed all reads to a maximum of 65 bp for mapping (chosen because it resulted in more similar mean lengths than did the 67-bp maximum length of CCD reads). Reads were mapped sequentially to a series of reference databases, allowing a defined number of mismatches (see Results) and normalized to the total number of reads in each library. We did not report counts with an additional normalization for reference length because the assembled contigs are generally fragments of larger molecules and, particularly for RNA viruses, are unlikely to include weakly expressed or noncoding regions. Since, for feasibility, the two sequenced samples were pooled from various sources as described, we cannot calculate technical or biological components of variance and thus make no statistical test of differential abundance between CCD+ and CCD−.

We performed an initial cull of all reads that mapped to known honey bee sequences, including the reference genome version Amel_4.0 [29] (GenBank accession PRJNA13343), GenBank accessions of mitochondrial and ribosomal sequence of that species (NC_001566, AY703484, AY703551), and contigs derived from this study that were BLASTN matches to the previous. Reads were then mapped to a database containing 1) representative GenBank accessions of known honey bee viruses: IAPV (NC_009025.1), KBV (NC_004807.1), acute bee paralysis virus (ABPV, NC_002548.1), chronic bee paralysis virus (CBPV, NC_010711.1 and NC_010712.1), DWV (NC_004830.2), BQCV (NC_003784.1), sacbrood virus (SBV, NC_002066.1), and slow bee paralysis virus (NC_014137.1); 2) whole genome sequences of eukaryotic pathogens and commensals, specifically the fungi *Ascosphaera apis* [30] (PRJNA17285) and *Saccharomyces cerevisiae* [31] (PRJNA128), the microsporidia *N. ceranae* [32] (PRJNA48321) and *N. apis* (Y.-P. Chen, unpublished data), the trypanosome *Crithidia mellificae* (R.S. Schwarz, unpublished data), the mite *Varroa destructor* [33] (PRJNA33465); and 3) GenBank accessions of bacterial ribosomal sequence that were classified as the dominant gut phylotypes by [20] (accessions listed below). After culling reads that matched this database, the remaining reads were mapped to the assembled contigs themselves. Residual reads were then mapped to ribosomal sequence of the SILVA database [34]. This last mapping was done only to identify the number of residual reads that were recognizably ribosomal, not to identify their taxonomic source (for which there is little power with short reads). After this first pass, unmatched reads were re-assembled with Velvet in a manner analogous to the original assembly, but with hash lengths of 23, 37, and 51, respectively. The resulting contigs were annotated with BLAST in the same manner as above and the mapping procedure re-iterated to produce the final read counts. After all Bowtie mapping steps, we used Mega BLAST to the genome and gene set of honey bee to identify residual bee sequence. Reads mapping to the Kakugo variant of DWV [35] were not treated separately due to the high nucleotide identity between the two. In contrast, Varroa destructor virus 1 [36] is more distinct from DWV, but only a single read mapped to this reference, such that we chose for simplicity to combine this read with the DWV counts.

The goals of the present study with respect to the honey bee microbiome were to further characterize what species were present and to identify any changes in species distributions between CCD− and CCD+ that are suggestive of physiological state or pathogenicity. However, this study was not designed specifically for metagenomic analysis of microbial community structure or gene content. It is inherently difficult to assemble short ribosomal reads from a diverse pool into contigs of sufficient length for phylogenetic assessment, and protein-coding transcripts from a source other than honey bee are expected to be poorly represented in total RNA. Uneven representation of phylogenetic groups in public databases and non-uniform criteria for their annotation are other sources of bias limiting our ability to accurately classify ribosomal sequence. There may also be unknown biological or methodological biases, such as variation among organisms in intrinsic expression level or the efficiency of RNA extraction. Relative abundances *within* a sample should therefore be treated with caution. However, there is no reason these obstacles to microbiotic classification should generate artifactual differences *between* equivalently prepared samples, especially in light of the relatively simple and stable community of honey bee gut bacteria [20]. We therefore mapped reads to GenBank accessions representative of this microbiota that were drawn from the phylogenetic analysis of [20] (AJ971849, AJ971850, AJ971857, AY370183–AY370186, AY370188, AY370191, AY370192, DQ837604, DQ837605, DQ837611, DQ837616, DQ837617, DQ837622–DQ837626, DQ837632–DQ837634, DQ837636, EF187232, EF187235–EF187237, EF187240, EF187242, EF187244, EF187250, EU055544, HM107876, HM108310, HM108312, HM108315, HM108316, HM108318, HM108324, HM108330, HM108332, HM108334, HM108335, HM108337, HM108346, HM108542, HM108563, HM111870, HM111875, HM111880, HM111883, HM111887, HM111901, HM111923, HM111924, HM111973, HM111977, HM112025, HM112033, HM112038, HM112042, HM112050, HM112068, HM112094, HM112104, HM112118, HM112130, HM112858, HM112866, HM113259, HM113300), as well as to contigs of our assembly that were considered bacterial based on BLAST match or the Classifier tool of the RDP database [27].

### Characterization of Novel Virus Candidates

Two groups of novel viruses were identified in this study (see Results) for which additional documentation was performed. One group had protein-level similarity to CBPV, now known to be variants of the recently described Lake Sinai Viruses [19], whereas the other group had protein-level similarity to members of the Partitiviridae. We sequenced PCR amplicons of several hundred base pairs to confirm the assembled sequences, for one LSV-related contig and for two partitivirus contigs. We used the following primer pairs: LSV, CATCGCAAATAGGCTGAGCA (forward) and CTCCTGGGTTGGCCTCACTA (reverse); Partitivirus1, TGAAGTCATGGATTGTAGTCTCGCT (forward) and CATCTGGTATGCCATGGTCTC (reverse), Partitivirus2, AGTCAAGCATCCGTGTTCATTC (forward) and TCGTGATCTGTTACCATCAGACTG (reverse). These amplicons all matched their predicted products and were deposited in GenBank as accessions JF732913–732915.

## Results

### Incidence, Abundance, and Covariance of Honey Bee Pathogens in CCD

CCD colonies showed moderately higher incidences of pathogens (**Table 1**) than non-CCD colonies. For all nine targets, the proportion of positive colonies was higher among CCD colonies than non-CCD colonies, although only DWV, KBV, and *N. apis* were significant at α = 0.05. Proportions of each target species were highly correlated between the two classes of hive (r = 0.97), indicating that common pathogens were common in both CCD and non-CCD hives and rarer pathogens were also rare in both. Concordant with the increased number of positive colonies, the mean number of pathogens present per colony was 4.8 (SE = 0.23) in CCD colonies and 3.6 (SE = 0.23) in non-CCD colonies, a significant difference of means (p = 0.004).

In addition to increased incidence of pathogens, CCD colonies also had higher loads of those pathogens, as measured by qPCR (i.e., a significantly lower mean ΔC_T_; **Table 2**). CCD colonies showed higher levels of the viruses BQCV, DWV, KBV, and ABPV as well as the gut parasites *N. apis* and *C. mellificae*. *N. apis* loads were over 20-fold higher in bees from CCD colonies than non-CCD colonies. In contrast to previous work [15], neither IAPV nor *N. ceranae* levels were significantly higher in CCD colonies.

**Table 2:** Contrasts in honey-bee pathogen abundance by colony status.

	All CCD colonies vs. all non-CCD colonies				Weak vs. strong colonies in non-CCD apiaries			
Target	ΔΔC_T_	SE	P-value	Fold change	ΔΔC_T_	SE	P-value	Fold change
ABPV	+2.23	0.96	0.02	4.57	+0.84	0.87	0.34	1.77
BQCV	+2.81	1.08	0.01	6.67	−1.70	1.59	0.29	0.32
DWV	+3.90	1.15	<0.01	14.26	−0.07	0.61	0.96	0.95
IAPV	+0.22	0.60	0.72	1.15	+1.64	0.98	0.11	2.83
KBV	+2.58	0.78	<0.01	5.49	+0.67	0.67	0.32	1.56
SBV	+0.28	0.64	0.66	1.27	+0.36	1.52	0.81	1.36
*N. ceranae*	+1.19	1.22	0.33	1.85	+1.70	1.59	0.29	2.41
*N. apis*	+3.94	1.07	<0.01	20.97	−0.03	0.14	0.81	0.98
*Crithidia*	+2.62	1.12	0.02	6.15	−0.79	1.95	0.69	0.58

The difference in mean ΔC_T_ values (normalized threshold cycle in qPCR reactions) was compared by ANOVA for two non-independent contrasts: all CCD colonies (n = 61) versus all non-CCD colonies (n = 63), and weak (n = 15) versus strong (n = 29) colonies in non-CCD apiaries. Weak colonies had six or fewer frames of bees and strong colonies had seven or more frames (see Materials and Methods). Non-CCD colonies include both sympatric and allopatric colonies, which were combined for increased statistical power. ΔΔ is the mean Δof the non-CCD population minus the mean Δvalue of the CCD population. Thus, positive numbers represent a decrease in mean threshold cycle and an increase in pathogen abundance. Fold change between categories is calculated as (1+ primer efficiency) ^ΔΔC^_T_. SE = standard error of population mean ΔC_T_; P-value = probability of equal mean ΔC_T_ by ANOVA (i.e., that the true ΔΔC_T_ = 0); ABPV = acute bee paralysis virus; DWV = deformed wing virus; SBV = sacbrood virus; BQCV = black queen cell virus; IAPV = Israeli acute paralysis virus; KBV = Kashmir bee virus.

To determine whether increased pathogen loads were dependent on CCD diagnosis or were merely characteristic of weak colonies in general, we also contrasted pathogen loads in ‘strong’ non-CCD colonies, with seven or more frames of bees, with those in ‘weak’ non-CCD colonies, with six or fewer frames. No pathogen had higher loads in ‘weak’ colonies relative to ‘strong’ (**Table 2**), indicating that higher pathogen loads is a hallmark of CCD rather than of a small colony *per se*.

**Figure 1:**
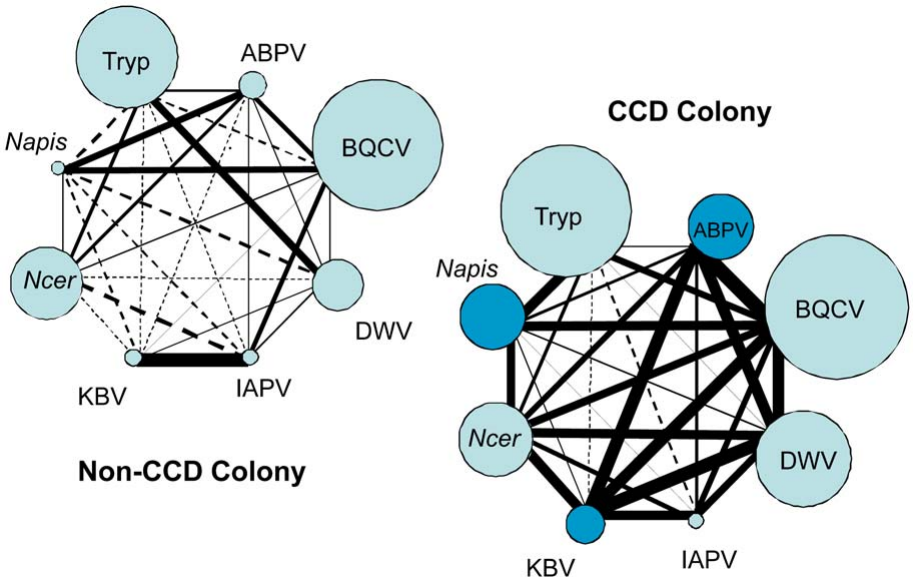
Graphical representation of pairwise correlations between pathogen abundance in CCD and non-CCD colonies. The thickness of lines is scaled to the Spearman’s rho correlation coefficient for each pair, the values of which are given in Table 3.

Covariation in abundance across different pathogen species was widespread in CCD colonies but rare in non-CCD colonies (**Table 3**). For CCD colonies, 11 of 36 pathogen pairs had significantly positively correlated ΔCT at a Bonferroni-corrected p<0.01. The RNA viruses ABPV, BQCV, DWV, and KBV were predominant in the list of significant pairwise interactions. **Figure 1** illustrates these “webs” of pathogen correlations in CCD compared with non-CCD colonies by linking each pair with a line the thickness of which is scaled to the correlation coefficient. SBV is not included in **Figure 1** because it was not significantly correlated with any other pathogen, perhaps because it replicates in larvae and adults are only carriers (although see below).

**Table 3:** Correlations of pathogen abundance within different colony types.

		CCD (n = 61)		Non-CCD (n = 63)		Non-CCD, sympatric (n = 37)		Non-CCD, non-sympatric (n = 26)	
Pathogen pair		Correlation	P-value	Correlation	P-value	Correlation	P-value	Correlation	P-value
BQCV	ABPV	0.606	<0.001*	0.135	0.291	0.214	0.204	0.018	0.928
DWV	ABPV	0.508	<0.001*	0.005	0.968	−0.018	0.914	0.035	0.863
KBV	ABPV	0.506	<0.001*	−0.003	0.981	0.029	0.865	−0.115	0.568
KBV	DWV	0.520	<0.001*	0.049	0.705	0.094	0.580	−0.021	0.918
KBV	IAPV	0.527	<0.001*	0.446	<0.001*	0.667	<0.001*	−0.083	0.681
DWV	BQCV	0.492	<0.001*	0.020	0.876	0.054	0.752	−0.037	0.854
KBV	BQCV	0.460	<0.001*	0.034	0.793	0.176	0.298	−0.295	0.135
*Crithidia*	NA	0.465	<0.001*	−0.110	0.391	−0.194	0.251	−0.115	0.568
NC	KBV	0.390	0.002*	0.004	0.974	0.084	0.621	−0.082	0.684
NC	DWV	0.361	0.004*	−0.038	0.769	0.142	0.401	−0.269	0.174
NA	BQCV	0.353	0.005*	0.289	0.022	0.361	0.028	0.140	0.485
NC	SBV	0.305	0.016	0.276	0.028	0.121	0.484	0.334	0.089
NA	NC	0.301	0.018	0.095	0.459	0.198	0.240	−0.163	0.415
NC	BQCV	0.278	0.030	0.130	0.310	0.119	0.482	0.126	0.533
IAPV	BQCV	0.273	0.033	0.195	0.126	0.310	0.062	−0.235	0.239
IAPV	DWV	0.233	0.071	0.120	0.350	0.099	0.562	0.181	0.367
SBV	KBV	0.228	0.074	−0.086	0.501	0.007	0.968	−0.145	0.470
*Crithidia*	BQCV	0.223	0.084	0.030	0.814	0.024	0.890	0.225	0.260
SBV	BQCV	0.212	0.098	0.088	0.495	−0.013	0.941	0.157	0.434
NC	ABPV	0.192	0.137	0.118	0.358	0.095	0.576	−0.044	0.829
NA	SBV	0.139	0.283	−0.080	0.534	−0.066	0.701	−0.089	0.661
SBV	DWV	0.151	0.240	0.099	0.440	0.222	0.192	0.070	0.727
NA	ABPV	0.146	0.260	0.284	0.024	0.259	0.122	0.353	0.071
SBV	ABPV	0.141	0.273	−0.012	0.929	0.314	0.062	−0.275	0.165
IAPV	ABPV	0.115	0.377	0.052	0.684	0.026	0.880	−0.023	0.908
*Crithidia*	IAPV	−0.096	0.461	−0.080	0.532	−0.174	0.304	0.050	0.806
NC	IAPV	0.093	0.478	−0.066	0.609	−0.185	0.272	0.240	0.228
*Crithidia*	ABPV	0.091	0.484	0.031	0.810	−0.048	0.778	−0.037	0.854
*Crithidia*	NC	0.088	0.499	0.215	0.091	0.402	0.014	0.084	0.678
NA	DWV	0.081	0.537	−0.167	0.191	−0.214	0.202	−0.120	0.552
SBV	IAPV	0.073	0.572	0.090	0.483	−0.106	0.538	0.511	0.007*
*Crithidia*	KBV	−0.056	0.666	−0.036	0.779	0.014	0.935	−0.154	0.442
*Crithidia*	SBV	0.053	0.683	0.160	0.209	0.032	0.852	0.398	0.040
NA	KBV	−0.035	0.789	−0.094	0.465	−0.125	0.460	−0.063	0.755
NA	IAPV	−0.013	0.920	−0.084	0.515	−0.121	0.477	−0.051	0.802
*Crithidia*	DWV	−0.000	0.999	0.273	0.031	0.390	0.017	−0.044	0.829

The number of colonies for each category is shown in parentheses. Sympatric non-CCD colonies are those that occurred in the same apiaries as CCD colonies, whereas non-sympatric colonies were sampled from different locations or in different years, or both, and thus were far removed from any diagnosed cases of CCD. The distinction is made because it was not possible to follow non-CCD colonies after sampling to determine if any subsequently experienced CCD. ABPV = acute bee paralysis virus; DWV = deformed wing virus; SBV = sacbrood virus; BQCV = black queen cell virus; IAPV = Israeli acute paralysis virus; KBV = Kashmir bee virus. Asterisk indicates a significant comparison after Bonferroni correction for multiple tests.

Samples from geographically distant sources had different pathogen complements. Colonies in the western U.S. tended to show higher incidences of pathogens (**Fig. S1,** panel A) than did colonies sampled at the same time in the eastern U.S. Both *N. apis* and ABPV were far more common in western colonies (**Fig. S1,** panel B), while IAPV trended higher in eastern colonies. KBV was the only pathogen to show higher abundance in CCD colonies in both eastern and western samples (**Fig. S1,** panel B).

Covariance in pathogen abundance was also observed at the level of individual bees drawn from colonies located in a CCD-free apiary (**Table 4**). These colonies were known to be infected with *N. ceranae* but were otherwise strong colonies. Six viruses and both *Nosema* species were measured by qPCR, although in these colonies CBPV was present and KBV was not. *Crithidia* levels were not measured in this cohort. Of the 28 pairwise correlations, 13 were significantly positive after Bonferroni correction. Even species likely to be in direct competition, such as *N. ceranae* and *N. apis*, which both reproduce in the gut epithelium, were positively correlated. Although the complement of pathogens in this single apiary differed somewhat from the colony-level survey as a whole, the data show that statistical interactions among pathogens scale to the level at which biological synergism is expected to be manifested, and that they are not secondary to some other CCD-associated variable.

**Table 4:** Pearson correlation coefficients of pathogen abundance within randomly sampled individual worker bees from colonies with an observable *Nosema* infection but no characteristics of CCD.

Pathogen pair		Correlation	Number ofco-infected bees	P-value
CBPV	ABPV	0.547	66	<0.0001*
DWV	NA	0.604	45	<0.0001*
DWV	NC	0.639	35	<0.0001*
DWV	CBPV	0.500	57	<0.0001*
SBV	ABPV	0.528	71	<0.0001*
SBV	CBPV	0.538	67	<0.0001*
DWV	BQCV	0.411	62	0.001*
NC	NA	0.482	34	0.004*
SBV	NC	0.427	42	0.005*
BQCV	NC	0.410	41	0.008*
CBPV	BQCV	0.322	63	0.010*
CBPV	NC	0.381	40	0.015*
IAPV	CBPV	0.283	69	0.018*
IAPV	ABPV	0.238	74	0.041
ABPV	NC	0.304	43	0.048
DWV	ABPV	0.248	62	0.052
BQCV	ABPV	0.219	68	0.073
SBV	DWV	0.229	62	0.074
SBV	BQCV	0.200	68	0.103
SBV	IAPV	0.168	74	0.153
BQCV	NA	0.181	49	0.214
IAPV	NA	−0.159	55	0.247
SBV	NA	0.159	53	0.257
IAPV	BQCV	0.042	71	0.731
CBPV	NA	0.039	49	0.792
IAPV	NC	−0.022	44	0.887
ABPV	NA	−0.009	55	0.950
IAPV	DWV	−0.001	65	0.991

These samples tested whether pathogen covariation occurred at the level of individual *Nosema*-exposed bees, outside of a CCD context. N equals the number of co-infected bees upon which the correlation is calculated for each pathogen pair, out of a total of 77 bees tested. CBPV = Chronic bee paralysis virus; ABPV = acute bee paralysis virus; DWV = deformed wing virus; SBV = sacbrood virus; BQCV = black queen cell virus; IAPV = Israeli acute paralysis virus; NC = *Nosema ceranae*; NA = *Nosema apis*. Asterisk indicates a significant comparison after Bonferroni correction for multiple tests.

### Metagenomic Analysis of RNA Sequences

Deep sequencing of RNA was performed primarily as a metagenomic strategy to identify novel pathogens that may be associated with CCD. The data were evaluated in two ways, first by annotating assembled contigs and then by classifying each sequence read according to the reference sequence they best matched, if any (see Materials and Methods). After quality-trimming, there were 19.28 million Illumina sequence reads for the non-CCD sample and 41.95 million reads for the CCD sample (counting paired reads separately). Our combined assembly of the two sets of reads produced 2,413 contigs with an N50 contig length of 436 bp. Contig sequences are given in **File S2**. **File S3** contains a spreadsheet of BLAST matches (expectation <1E−10). The number of reads mapping to sequential reference sequences (see Materials and Methods) at each step is summarized in **Table 5**.

**Table 5:** Assignment of Illumina sequencing reads to bins.

				CCD−			CCD+		
Mapping step	Reference	Program	Match criterion	Starting reads (000s)	Mapped reads (000s)	Percent of total reads mapping	Starting reads (000s)	Mapped reads (000s)	Percent of total reads mapping
1	Honey bee reference sequence	Bowtie	V = 2	19,276	16,921	87.8%	41,950	37,692	89.9%
2	Reference sequence for known eukaryotic, prokaryotic, and viral associates of honey bee	Bowtie	V = 2, best	2,355	195	1.0%	4,258	496	1.2%
3	Assembly contigs	Bowtie	V = 2, best	2,160	1,586	8.2%	3,762	2,992	7.1%
4	Silva LSU and SSU ribosomal databases (4/21/2010)	Bowtie	V = 3	574	116	0.6%	770	293	0.7%
5	NCBI Univec database	Mega BLAST	E <0.01	458	41	0.2%	477	11	0.03%
6	Honey bee reference sequence	Mega BLAST	E <0.01	417	63	0.32%	466	69	0.16%

Match criteria are the conditions under which an aligned read is considered a valid mapping. For Bowtie, this column shows the number of mismatches (the parameter ‘V’) and whether the match was required to be the best match in the reference database. For Mega BLAST, the minimum expectation of the match is shown.

The distribution of contigs by best BLAST match is shown in **Table 6**. As expected, the majority of contigs (1,683 or 70%) matched the genus *Apis*. Another 35 contigs had best matches to other insects, principally ribosomal sequence from the genus *Bombus* and other bees. These contigs are presumed to be *A. mellifera* alleles that diverge from the reference genome, rather than genuinely derived from another species. Smaller numbers of contigs were homologous to various pathogens included in the qPCR survey, such as *Crithidia*, *Nosema*, and most of the RNA viruses investigated. Surprisingly, twelve contigs homologous to *Varroa* ribosomal loci were identified; since these are relatively large ectoparasites that are readily removed, contamination of honey bee RNA by *Varroa* was not expected. However, the possibility that cells or RNA moieties are transferred to bees by feeding mites is suggested by the fact that other investigators have also found *Varroa* ribosomal sequence in *A. mellifera* deep-sequencing reads (e.g., GenBank accession HP469569 from a 454 transcriptome assembly).

**Table 6:** Best BLAST match of contigs assembled from deep sequencing of the CCD+ and CCD− cDNA libraries derived from pooled colony samples of total RNA.

Taxon	Number of contigs
*Apis mellifera*	1683
Other insect*	35
*Crithidia* **	10
*Nosema apis*	10
*Nosema ceranae*	8
*Varroa destructor*	12
Fungi	19
Plants	58
Uncultured eukaryote	7
Nematoda	1
Eubacteria	303
Black queen cell virus	5
Deformed wing virus	6
Israeli acute paralysis virus	9
Kashmri bee virus	2
Sacbrood virus	1
Lake Sinai Virus 1	25
Lake Sinai Virus 2	85
Partitiviridae	3
Tobacco Ring Virus	2
No match	129

* Best BLAST match was to ribosomal sequence of another insect species but contig is presumed to derive from *A. mellifera.*

** Includes the trypanosome genera *Leishmania* and *Leptomonas.*

Fifty-eight contigs were apparently of plant origin and presumably derive from consumed pollen, as has been observed in other studies (e.g., [20]). Fungi were the next most abundant group of eukaryotes, but none of the top BLAST matches to these contigs were known entomopathogens. Nine of 19 contigs were yeasts related to *Saccharomyces*/*Zygosaccharomyces* and six more were strong matches to other members of the *Saccharomycetaceae*, which is consistent with the known abundance of yeasts in the honey bee gut [37]. Three contigs had greatest similarity to the plant-pathogenic genera *Cronartium*, *Endocronartium*, and *Melamspora* and were likely associated with pollen. The remaining contig had greatest similarity to a common environmental fungus, *Myceliophthora thermophila*. No contigs had best BLAST matches to fungi related to *Penicillium* or *Aspergillus*, which have been reported to be present in honey-bee guts [37].

We identified 303 contigs that had bacterial best BLAST matches. Using the Classifier tool for 16S ribosomal loci, we could assign 67 of these contigs to bacterial orders with 80% bootstrap support (**Table 7**). The identified taxa were consistent with previous studies of the honey bee gut microbiome ([15], [20] and references therein), including a diversity of Lactobacillales and Enterobacteriales. The remaining contigs were either phylogenetically ambiguous at this confidence level or not 16S sequences. Among these unclassified contigs, 12 had strong BLASTN matches to the *Melissococcus plutonius* genome (GenBank accession AP012200), the bacterial pathogen underlying European foulbrood disease of honey bees (reviewed by [38]). This pathogen was modestly more abundant in CCD+ by read count (a log2 difference of +0.39, or an increase of 31%). The bacterial pathogen causing American foulbrood, *Paenibacillus larvae* [39], was also detected by read mapping, but was also only moderately more abundant in CCD+ (a log2 differential of +0.10, or a 7% increase). Four contigs had best BLAST matches to the bacterial genus *Arsenophonus*, which is known to occur as an intracellular symbiont in some insect species and has been reported in honey bee [40].

**Table 7:** Taxonomic distribution of cDNA contigs with homology to 16S ribosomal sequence, by bacterial order.

Class	Order	Number of 16S contigs	Log2 difference in read counts (CCD+/CCD−)
Actinobacteria	Bifidobacteriales	3	−0.82
Alphaproteobacteria	Rhizobiales	7	−1.47
Alphaproteobacteria	Rhodospirillales	6	−0.90
Bacilli (Firmicutes)	Clostridiales	5	+0.19
Bacilli (Firmicutes)	Lactobacillales	24	+0.29
Betaproteobacteria	Burkholderiales	1	N/A (3 reads in CCD−, 2 reads in CCD+)
Betaproteobacteria	Neisseriales	3	+0.32
Gammaproteobacteria	Enterobacteriales	13	+0.42
Gammaproteobacteria	Pseudomonadales	5	N/A (64 reads in CCD−, 1 read in CCD+)

Taxonomy was estimated by the RDP Classifier tool for all 303 contigs with bacterial best BLAST matches. Only the 67 contigs with a minimum 80% bootstrap support at the order level are included here. N/A = not applicable, due to a low number of mapped reads.

**Figure 2:**
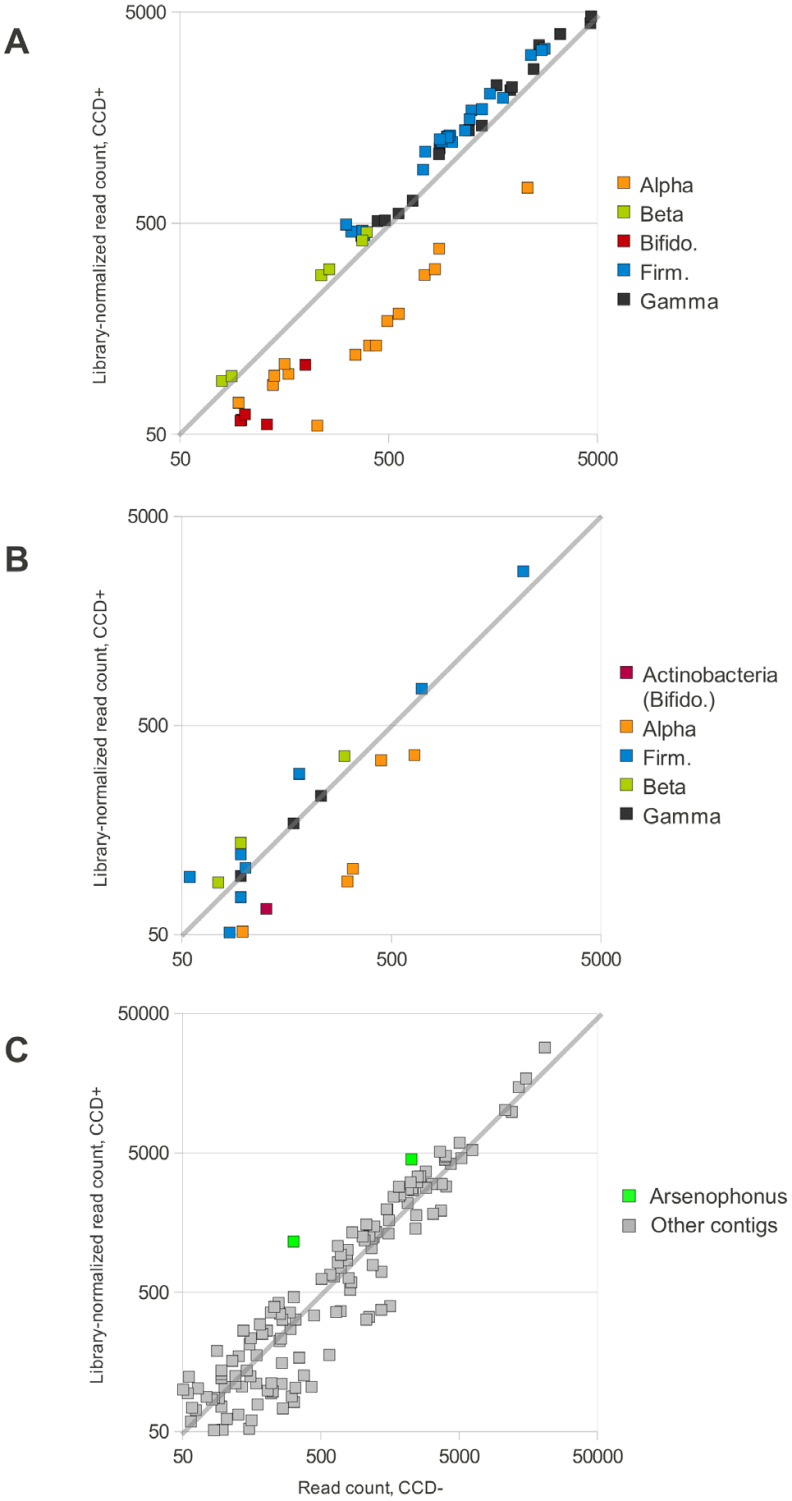
Change in abundance of bacterial taxa inferred from mapping of Illumina reads. In all three panels, the horizontal axis is the number of reads mapping to each reference in the CCD− sample and the vertical axis is reads mapped in CCD+, adjusted for library size. The gray diagonal line in each panel demarcates equal representation in the two samples, and the axes are log10 scale. Only references with normalized read counts greater than 50 in each sample are displayed. **A.** Read counts for 72 GenBank accessions that are representative of the major gut microbial phylotypes of the honey bee. The accessions are drawn from Fig. S1 of [20] and are listed in Materials and Methods. Each accession is color-coded by taxonomy, following the phylotypes of [20]: Alpha = the alpha-proteobacteria clusters Alpha1, Alpha2.1, and Alpha2.2; Beta = beta-proteobacteria cluster, Gamma = the gamma-proteobacteria clusters Gamma1 and Gamma2, Bifido. = *Bifidobacteria*, and Firm. = the firmicutes clusters Firm4 and Firm5. **B.** Read counts of contigs in **File S3** that were assigned to bacterial phyla using the Classifier tool [27]. All three actinobacteria contigs belonged to the genus *Bifidobacteria* based on high Classifier bootstrap support at the genus level (**File S4**) and best BLAST match (**File S3**). Other contigs are color-coded by phylum: Alpha = alpha-proteobacteria, Beta = beta-proteobacteria, Firm = firmicutes, and Gamma = gamma-proteobacteria. **C**. Read counts of all contigs with bacterial BLAST matches. A more diffuse but still bimodal distribution of relative change in read counts is apparent. The two contigs that show the greatest increase in CCD+ relative to other contigs (highlighted in green) both have best BLAST matches to the genus *Arsenophonus* with an expectation at least four orders of magnitude lower than the next closest taxon, but the maximum identity of these matches is only 90%.

The gut bacteria of honey bees can be clustered by 16S ribosomal sequence into a relatively small number of distinct phylotypes that are numerically predominant [20]. To examine how these major bacterial groups vary between CCD− and CCD+, we compared the relative abundance of reads mapping to 72 GenBank accessions that are representative of these phylotypes (see Materials and Methods). **Figure 2A** shows a strong and consistent pattern in which accessions representative of the Alpha1, Alpha2.1, Alpha2.2, and Bifidobacterium phylotypes of [20] are reduced in CCD+, with log2 differences in the range of −0.5 to −1.5. In contrast, the Betaproteobacteria, Firmicutes, and Gammaproteobacteria phylotypes are consistently increased in CCD+, by more moderate amounts. Since these phylotypes are numerically dominant among honey-bee gut bacteria, changes in their numbers are likely to be autocorrelated, such that the opposing direction of change in these two groups of taxa may well reflect a common underlying cause.

Although short sequence reads lack sufficient resolution for taxonomic quantification when many taxa are plausible matches, in this case the majority of bacterial reads are expected to map to only one phylotype. Furthermore, the results are consistent when mapped either to the 16S references or the assembled contigs. **Fig. 2A** shows that the differential read counts are consistent among the different accessions that constitute each phylotype and are not driven by individual outliers. The 67 contigs that were assigned by Classifier to a bacterial order exhibited a comparable deficit in CCD+ (**Fig. 2B**) for some alpha-proteobacteria as well as actinobacteria that are presumed to be *Bifidobacterium* based on Classifier output and BLAST match. Read mapping to all 303 bacterial contigs again suggests a bimodal distribution of change in relative abundance (**Fig. 2C**). Interestingly, two *Arsenophonus*-related contigs had the highest proportional increase in CCD+ among all contigs with moderate to high read counts (>1,000 mapped reads in either sample. These contigs are highlighted in **Fig. 2C**.

**Figure 3:**
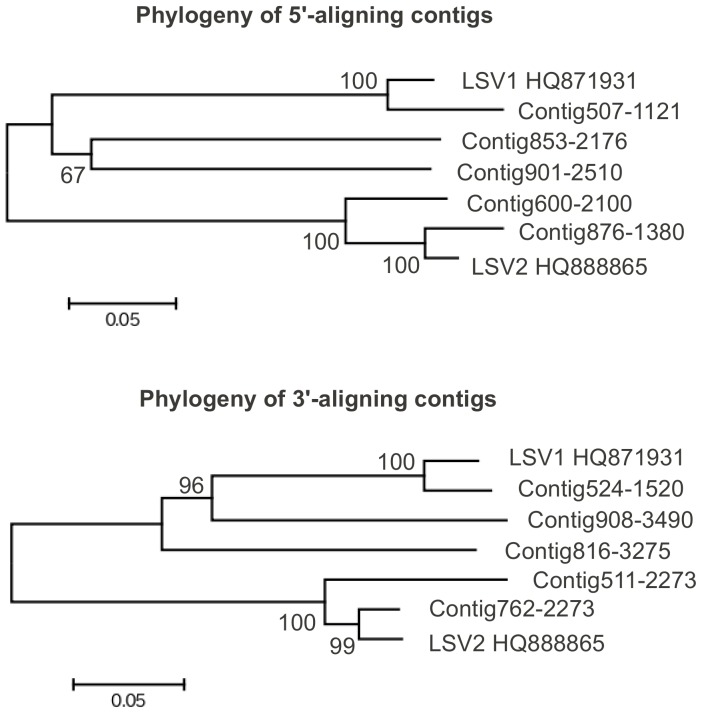
Phylogeny of contigs related to the Lake Sinai Viruses (LSV1 and LSV2). **A**. Phylogeny of the five longest 5′-aligning contigs with LSV1 and LSV2 (GenBank accessions HQ871931.1 and HQ888865.1) **B**. Phylogeny of the five longest 3′-aligning contigs with LSV1 and LSV2. The two trees have similar branch lengths and topologies, suggesting that a physical linkage between each 5′-aligning contig and a 3′-aligning contig.

**Figure 4:**
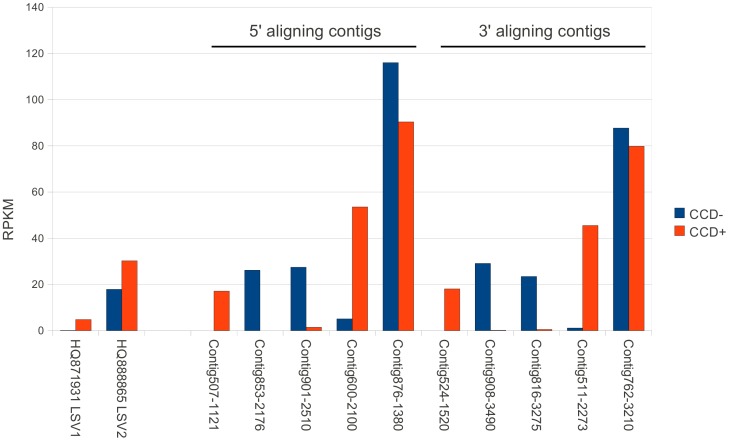
Relative abundance of LSV strains in CCD− and CCD+ samples. Contigs and accessions are the same as in **Figure 3**, with contigs aligning to the 5′ and 3′ regions, respectively, of LSV denoted as such. The frequency of mapped reads for each 5′ aligning contig is mirrored by that of a corresponding 3′ contig, suggesting physical linkage. Here read counts are normalized by contig length (reads per kilobase per million mapped reads, or RPKM) because the frequency of viral fragments of different lengths are being compared.

Several novel RNA virus sequences were identified in the assembled contigs. BLASTX matches to LSV1 or LSV2 [19] were found for 109 contigs, indicating that these recently identified viruses are widespread in the U.S. Interestingly, contigs or reads were not found that matched the reference genome of CBPV itself (GenBank accessions NC_010711 and NC_010712) [41]. Twelve of the contigs with homology to LSV were greater than 1 kb but none covered the full length of the coding sequence of LSV1 and LSV2. We therefore investigated the diversity of LSV sequences by constructing separate nucleotide phylogenies for the five longest contigs aligning with the 5′ end of these viruses (**Fig. 3A**) and the five longest contigs aligning with the 3′ end (**Fig. 3B**). The trees for these two groups of contigs are similar in branch lengths and topology, suggesting a one-to-one correspondence between each 5′-aligning contig and a 3′-aligning contig. This inference is further supported by the distribution of reads mapped to each of these contigs and to LSV1/LSV2 **(** **Fig. 4** **)**. That is, normalized read counts for each contig in **Fig. 3A** mirror those for a contig in **Fig. 3B** with a similar phylogenetic position (for example, Contig600 and Contig511). Additional sequencing of longer LSV clones beyond the scope of this study is needed to clarify the diversity of this viral taxon, but the weight of evidence suggests that multiple strains intermediate between LSV1 and LSV2 were present in the sampled colonies. Interestingly, while LSV2 and the contigs most closely related to it (Contig876 and Contig762) were comparatively even in abundance in CCD− and CCD+, LSV1 and the other LSV-related contigs showed pronounced deviations between the two samples (**Fig. 4**). This observation suggests a potential association between LSV strain and CCD status that merits further investigation.

**Figure 5:**
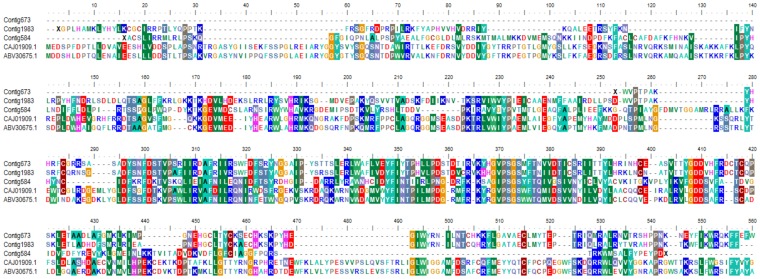
Sequence alignment of three contigs with BLASTX matches to the RDRP of *Penicillium stoloniferum virus S*, GenBank accessions CAJ01909.1 and AY156521.2. Shading at each position indicates amino-acid similarity among at least 50% of the residues, based on the BLOSUM62 matrix. Alignment performed with ClustalW using default settings.

An additional three contigs were identified with BLASTX matches (expectation <1.0E-10) to the RNA-dependent RNA polymerase (RDRP) of viruses in the Partitiviridae, which are double-stranded RNA viruses with segmented genomes [42]. The level of amino-acid sequence identity of these matches was 30–36% (**Fig. 5**) and matches to other regions, such as capsid-encoding genes, were not detected in our assembly. As replicating partitiviruses have been identified to date only from fungi and plants [42], it seems probable that these viral sequences derive from fungi or pollen present in the gut rather than honey bee tissue. They are nonetheless noteworthy in that they were found almost exclusively in CCD+ (1733 reads versus 9 in CCD−, or a normalized, log2 differential of +6.5).

Finally, two short, low-coverage contigs were near exact matches to Tobacco Ringspot Virus, a single-strand RNA virus of the Picornavirales. We are not aware of any published reports of bees infected by this virus and assume that it is associated with pollen.

We did not detect any known DNA virus transcripts in the assembled contigs using BLASTX (best match with an expectation <1.0E−10). However, as it has been recently argued that an insect iridescent virus (IIV) contributes to CCD [43], we performed an additional search for this virus. We identified all ORFs of 20 amino-acids or more from all unmapped reads and used BLASTP to compare these to all GenBank protein accessions of IIV3 and IIV6, the most representative taxa proposed for the putative honeybee IIV [43]. Only a single read in each sample had a match to these reference sequences, and in both cases those reads were better matches to unrelated viral proteins. We conclude that if an IIV was present in these samples, it was not transcriptionally active at detectable levels.

### Comparison of Read Mapping to qPCR Estimates of Pathogen Loads

A second objective of RNA sequencing was to corroborate the qPCR estimates of pathogen loads. To do this, we converted the fold change in pathogen abundance inferred from qPCR (**Table 2**) to “expected” log2 differentials in reads and compared these with actual log2 differentials in mapped reads. There was general agreement between the two methods for seven of the nine targets (**Table 8**). For ABPV and SBV, reads were mapped from CCD+ but not CCD−, consistent with the qPCR results for these viruses, but preventing a quantitative estimate of relative change. On the other hand, there was a strong discrepancy in *Crithidia* abundance assessed by the two methods. The decline in trypanosome-mapped reads in CCD+ was similar regardless of whether a GenBank ribosomal accession (GU321196), assembled contigs (**Table 6**), or a draft whole-genome assembly of *C. mellificae* strain ATCC-30254 (R. Schwarz unpublished data) was used as the reference (results not shown). However, since another, highly divergent lineage of *Crithida* has been isolated and sequenced from infected honey bees (J. De Risi, unpublished data), it remains possible that the strong discrepancy between the two methods reflects an underlying genetic heterogeneity. A second major discrepancy was seen for IAPV abundance. Genotypic variation is an unlikely explanation in this case, however, as three primer pairs were used to detect this virus. Given that cDNA sequencing libraries were generated from RNA that had been pooled from many samples, and that viral abundances are typically skewed (i.e., a few samples have values much higher than the mean), even small stochastic errors at this stage could introduce nontrivial technical variation. IAPV was infrequent generally (**Table 1**), exacerbating this potential random error. Also, our more stringent requirement that three primer pairs amplify for an IAPV sample to be considered positive may have prevented low-level infections from being detected in the qPCR analysis, but would not have biased the sequencing pool. Despite the uncertainty regarding IAPV abundances, the data are at least consistent with there being no significant increase in IAPV incidence or abundance in our CCD samples, contrary to earlier work [15]. More work is needed to clarify the disagreement between methods for these two pathogens, but we do not believe it materially affects the conclusions of the study.

**Table 8:** Comparison of RNA target abundances in Illumina sequence reads compared with qPCR results.

Target	Expected log2 differential abundance in pool based on qPCR	Normalized log2 differential abundance of mapped reads	Comments
ABPV	+2.27	N/A	0 reads in CCD−, 8 reads in CCD+
BQCV	+2.89	+1.72	
DWV	+3.97	+2.14	
IAPV	+0.24	−2.85	The two measures are strongly discordant
KBV	+2.71	+5.12	2 reads in CCD−, 126 reads in CCD+
SBV	+0.22	N/A	0 reads in CCD−, 25 reads in CCD+
*Nosema apis*	+3.54	+4.10	
*Nosema ceranae*	+1.60	+0.87	
*Crithidia*	+2.62	−1.57	The two measures are strongly discordant

The expected log2 differential is taken as the log2 of fold change in CCD colonies from Table 2. For read counts, differential abundance = log2 (CCD+ reads) – log2 (CCD− reads) – log2 (total CCD+ reads/total CCD− reads). N/A indicates that the log2 difference in abundance could not be calculated because no reads matched in CCD−.

## Discussion

Colonies of the domesticated honey bee have been in decline in the United States for sixty years. This decline has been driven in part by economic forces, including the increased costs of disease management [10]. Nevertheless, honey bee colony losses in the U.S. have reached new highs in the past several years, exceeding 30% country-wide during the vulnerable winter period (an absolute rate of 400,000+ colonies each winter in the United States alone) [10]. Parasites and infectious agents have been posited to play a role in CCD, a syndrome tied to many of these overwinter colony losses. RNA viruses and microsporidia have been implicated in past studies [15], [16], but no single pathogen has been identified that is consistently associated with collapse. An emerging hypothesis to explain these findings is that interactions among multiple subclinical infections can lead to the rapid depletion of adult workers that characterizes CCD. Alternatively, CCD as operationally defined could conflate unrelated diseases that produce similar phenotypes, thereby confounding studies of the underlying causes. More extensive studies of biotic correlates with CCD have been needed to clarify these issues.

Here we have presented a retrospective study of pathogen incidence, abundance, and covariance in a large, geographically diverse sample. Our results revealed an increase in pathogen loads and extensive pathogen covariance in CCD colonies that were not observed in weak colonies generally. No single pathogen was uniformly associated with CCD, however, consistent with the body of data on the subject. For example, levels of the microsporidian pathogen *N. apis* were more than an order of magnitude higher in CCD samples overall, but it was completely undetected in eastern cases of CCD. Its congener *N. ceranae* was widespread but not significantly increased in CCD colonies. However, positive correlations between *N. ceranae* and other pathogens were observed at both the colony and individual levels. In CCD colonies, *N. ceranae* loads were significantly correlated with levels of DWV and KBV. Individual bees from *Nosema*-infected colonies that were otherwise strong showed positive correlations between the loads of *N. ceranae* they carried and the level of co-infecting DWV, SBV, CBPV, BQCV, and *N. apis*, demonstrating that these interactions can occur independently of CCD status. These results support other studies that have linked *Nosema* infection with increased susceptibility to other pathogens [15], [16]. The strong association of *N. apis* with colony collapse in this study is somewhat unexpected given the apparent decline in its geographic distribution [18] and data indicating a more detrimental effect of *N. ceranae* infection [16], although work in a colder climate found little impact by either species [44].

Most of the known RNA viruses quantified in this study were significantly more abundant in CCD colonies. This general pattern of increased viral loads is consistent with other published data [45], [46]. However, given the strong association between IAPV and CCD in one prior survey [15], it is puzzling that we found no positive association between the presence or infection load of this virus and CCD. Our detection strategy and sampling approach were similar to but somewhat broader than the earlier survey. Specifically, we had a stronger focus on the western U.S., where IAPV was generally scarce in both normal and collapsed colonies. While our qPCR and read count data conflicted regarding IAPV abundance in CCD colonies, we are not likely to be under-estimating its frequency with qPCR but rather may be over-estimating it. IAPV remains a bee pathogen of concern, however, given its worldwide distribution [47], [48], [49] and experimentally demonstrated association with honey bee mortality [47]. We did find strong correlations with disease for the closely related viruses ABPV and KBV, and as such the family Dicistroviridae remains linked to poor bee health.

We found no transcript evidence for an iridescent virus of honey bees. These DNA viruses were recently proposed to have an association with CCD based on proteomic work [43], a result that has since been strongly criticized on methodological grounds [50]. No nucleic acid sequence attributable to a honey bee IIV has been isolated, so a more definitive assessment is not possible with our data. However, our analyses imply that IIV, if present, is unlikely to be a major contributor to CCD in the geographic regions covered by this survey.

The gut microbiota play important roles in host health and nutrition [37], and our survey found evidence of a phylogenetically clustered shift in the honey bee bacterial community involving declines in *Bifidobacterium* and alpha-proteobacteria. Although short-read sequencing provides limited resolution of taxonomic groups, the coherence and magnitude of change in these taxa support their biological relevance. Since CCD colonies have a marked deficit of older workers, age structure *per se* could well contribute to the bacterial pattern observed. The apparent increased abundance in CCD of bacteria related to *Arsenophonus*, an endosymbiont genus identified in numerous insects, is an intriguing observation, but it is not yet clear how often colonies harbor these bacteria (they are not among the predominant phylotypes that have been identified by [20] and others). The phylogenetic relationship of these contigs with other described *Arsenophonus* remains to be clarified, but our results suggest a potential association with bee health that merits further investigation. Yeasts are also important components of the honey bee gut microflora [37] and we found ribosomal sequence related to *Saccharomyces* as expected (**Table 6**). However, we did not detect other fungi that have been reported in the honey bee gut, such as *Aspergillus* and *Penicillium* species, although their distributions are considered more erratic [37]. Metagenomic studies of the interactions among bacteria, fungi, and their host constitute an important future direction of apicultural research.

Honey bees play critical pollination roles in natural and managed ecosystems, and an understanding of the biological causes behind honey bee losses will enable improved management and breeding strategies aimed at improving bee health. Here we describe the most extensive survey to date of microbes associated with CCD colonies. We have decoupled otherwise weak colonies from those diagnosed with CCD and have shown that the latter colonies have substantially heavier pathogen loads (although whether this increase is a cause or an effect of CCD remains unknown). Via *de novo* transcript assembly, we have identified novel RNA viruses of potential importance to bee health that can now be characterized with controlled infections and molecular analyses. The diverse LSV sequences are of particular interest because outbreaks of the distantly related CBPV have been known to cause workers to die en masse away from the hive, albeit rarely [51].

### Future Directions

An inherent limitation of our approach is the unknown degree to which bees remaining in a CCD hive at the time of sampling can serve as indicators of the events leading to its decline and the physiological status of the missing bees. However, the greater abundance and covariance of pathogens in CCD hives are informative and must be explained by any proposed model of how CCD occurs. A definitive analysis of the causes of CCD will ultimately require its controlled replication through the experimental manipulation of the relevant variables. Given the complexity of natural systems and the number of potential variables, retrospective and prospective observational studies are necessary for narrowing hypotheses to a manageable number. Our results indicate several promising pairings for such tests, in particular, *Nosema* with the RNA viruses DWV, KBV, BQCV, or ABPV. If pathogen webs are indeed precipitators of colony collapse, future work must demonstrate how this occurs at the level of individual bees and the overall hive to produce a rapid loss of foragers without overt disease in the remaining bees. The apparent variation in pathogen distributions, including strain variation, also needs to be better described in order to 1) identify and investigate discrepancies between epidemiological and experimental data, and 2) better inform management and policy decisions, including the possibility of quarantine. Agrochemical exposure also needs to be more fully explored as a contributor to CCD, and we stress that our results do not speak for or against its role in colony loss.

## Supporting Information

Figure S1 **Differential microbial abundances for colonies sampled in the western and eastern United States.** A. Proportional abundance of discitroviruses (red), iflaviruses (blue), bacteria (green), microsporidia (pink) and trypanosome (orange) pathogens in non-CCD (n = 38) and CCD (n = 61) bee samples, as indicated by letter size. A = ABPV, I = IAPV, K = KBV, D = DWV, Q = BQCV, S = SBV, B = bacterial load, C = *Nosema ceranae*, P = *Nosema apis*, T = *Crithidia*. B. Mean relative abundances (ΔC_T_) of four viruses and two *Nosema* species in CCD and non-CCD colonies in the two geographic regions. For comparison, the values are scaled by adding a constant such that the minimum value of all samples is zero.
(TIF)

File S1 **Estimated efficiencies of qPCR reactions using primers, templates, and reaction conditions described in the text.**
(DOC)

File S2 **cDNA contigs resulting from our assembly of Illumina sequence reads.**
(TXT)

File S3 **Best BLAST matches for the assembled contigs.**
(XLS)

File S4 **Classifier** [**27**] **taxonomic assignments for contigs with at least 80% bootstrap support at the level of order.**
(XLS)
