# Supplementary figures and images for: Pathogen Webs in Collapsing Honey Bee Colonies
Source: PLoS One. 2012 Aug 21;7(8):e43562. doi: 10.1371/journal.pone.0043562 (PMC3424165; doi:10.1371/journal.pone.0043562)

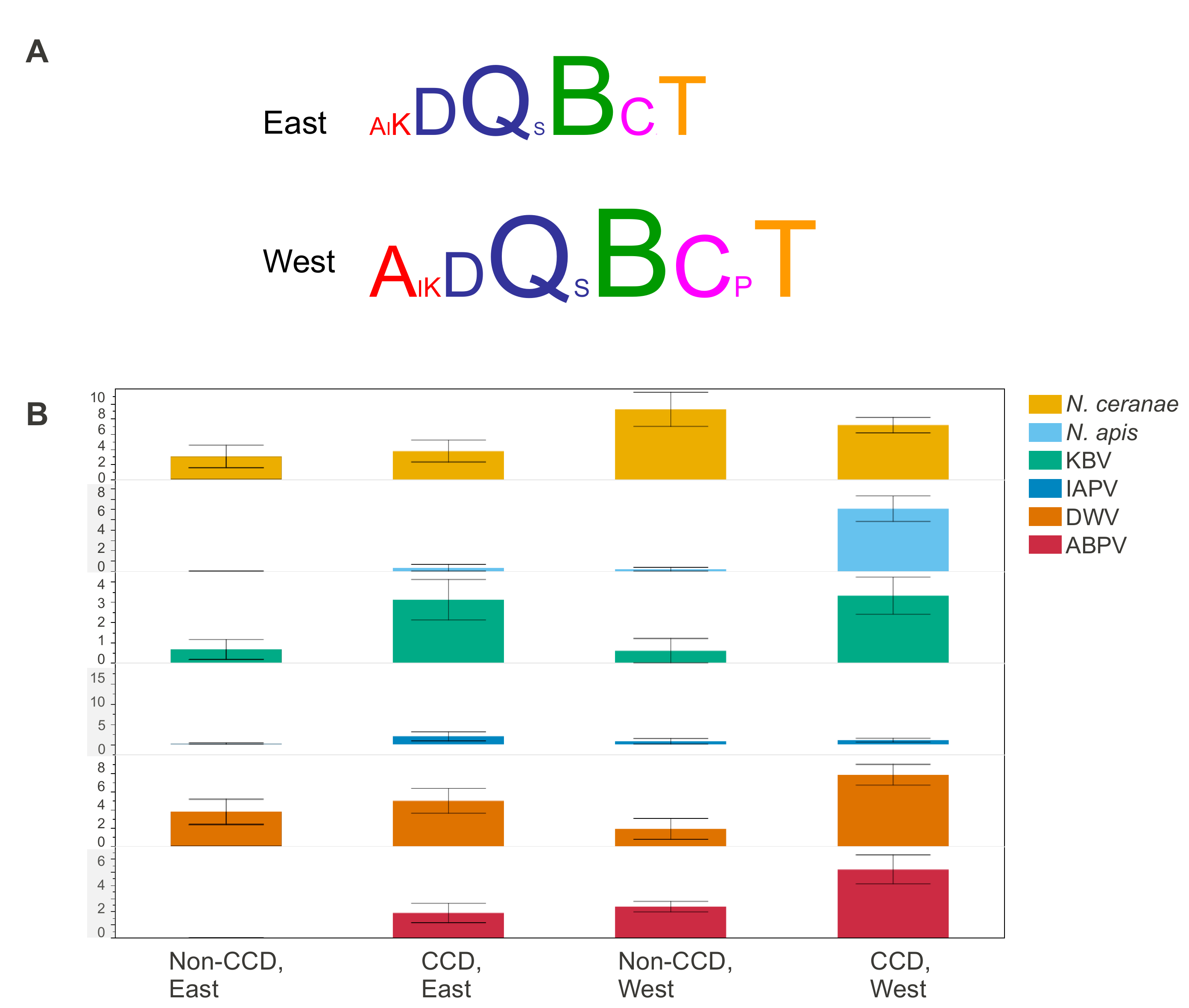

Supplement: Figure S1 — Differential microbial abundances for colonies sampled in the western and eastern United States. A. Proportional abundance of discitroviruses (red), iflaviruses (blue), bacteria (green), microsporidia (pink) and trypanosome (orange) pathogens in non-CCD (n = 38) and CCD (n = 61) bee samples, as indicated by letter size. A = ABPV, I = IAPV, K = KBV, D = DWV, Q = BQCV, S = SBV, B = bacterial load, C = Nosema ceranae, P = Nosema apis, T = Crithidia. B. Mean relative abundances (ΔCT) of four viruses and two Nosema species in CCD and non-CCD colonies in the two geographic regions. For comparison, the values are scaled by adding a constant such that the minimum value of all samples is zero. (TIF) [file pone.0043562.s001.tif]
