# Supplementary material for: Pathogen Webs in Collapsing Honey Bee Colonies
Source: PLoS One. 2012 Aug 21;7(8):e43562. doi: 10.1371/journal.pone.0043562 (PMC3424165; doi:10.1371/journal.pone.0043562)
Supplement: File S1 — Estimated efficiencies of qPCR reactions using primers, templates, and reaction conditions described in the text. (DOC) [file pone.0043562.s002.doc]

**Supporting Information File 1.** Estimated primer efficiencies for calculation of fold change in pathogen abundance by qPCR. Primer sequences are cited in the Materials and Methods. Three primers were used to detect IAPV, but primer IAPV-F1a was used for quantification.

| Type | Species | Designation | Efficiency by 10X dilution series analysis |
| --- | --- | --- | --- |
| Pathogen | Acute bee paralysis virus | ABPV | 0.98 |
| Pathogen | Black queen cell virus | BQCV | 0.96 |
| Pathogen | Chronic bee paralysis virus | CBPV | 0.98 |
| Pathogen | Deformed wing virus | DWV | 0.98 |
| Pathogen | Israeli acute paralysis virus | IAPV-F1a | 0.88 |
| Pathogen | Kashmir bee virus | KBV | 0.94 |
| Pathogen | Nosema apis | NA | 1.16 |
| Pathogen | Nosema ceranae | NC | 0.69 |
| Pathogen | Sacbrood virus | SBV | 1.37 |
| Pathogen | Crithidia mellificae | Crithidia | 1.00 |
| Normalization reference | Apis mellifera | Actin | 1.07 |
| Normalization reference | Apis mellifera | MGST | 0.98 |
| Normalization reference | Apis mellifera | RPS5 | 1.03 |
